# Etiopathogenesis of benign prostatic hypeprlasia

**DOI:** 10.4103/0970-1591.56179

**Published:** 2009

**Authors:** Jie Tang, JingChun Yang

**Affiliations:** Department of Ultrasound, Chinese People's Liberation Army General Hospital, P. R. China

**Keywords:** Benign prostatic hyperplasia, etiopathogenesis

## Abstract

Benign prostatic hyperplasia (BPH) is the most common condition affecting men older than 50 years of age. It affects about 10 percent of men under the age of 40, and increases to about 80 percent by 80 years of age. BPH is a hyperplastic process of the fibromuscular stromal and glandular epithelial elements of the prostate. Aging and the presence of the functional testes are the two established risk factors for the development of BPH. The etiopathogenesis of BPH is still largely unresolved, but multiple partially overlapping and complementary theories have been proposed, all of which seem to be operative at least to some extent. This review is focused on recent progress in this area and on the growing consensus for the important mechanisms underlying the etiology and pathogenesis of BPH.

## INTRODUCTION

Benign prostatic hyperplasia (BPH) is the most common condition affecting men older than 50 years of age. It affects about 10 percent of men under the age of 40, and increases to about 80 percent by 80 years of age. BPH is a hyperplastic process of the fibromuscular stromal and glandular epithelial elements of the prostate. Aging and the presence of the functional testes are the two established risk factors for the development of BPH. Although not a life-threatening condition, BPH can significantly affect quality of life. BPH manifests clinically with lower urinary tract symptoms (LUTS) and may be associated with sexual dysfunction.[1]

The etiopathogenesis of BPH is still largely unresolved, but multiple partially overlapping and complementary theories have been proposed, all of which seem to be operative at least to some extent. This review is focused on recent progress in this area and on the growing consensus for the important mechanisms underlying the etiology and pathogenesis of BPH.

## ROLE OF ANDROGENS AND ESTROGENS IN BPH DEVELOPMENT

The prostate consists of a network of glandular elements embedded in stroma, with androgen being the most important factor for prostatic growth. Androgens elicit their morphogenetic effects by acting through androgen receptors (ARs) in urogenital sinus mesenchyme (UGM), which induces prostatic epithelial development. In adulthood, reciprocal homeostatic stromal-epithelial interactions maintain functional differentiation and growth-quiescence. It is generally held that androgens are not only required for normal function of the prostate gland but also have been implicated in prostate disease as well.[2]

The breakthrough discovery that DHT is the active factor in the prostate is the rationale for the use of 5a-reductase inhibitors in the treatment of BPH. DHT-mediated effects within the stromal compartment produce growth-stimulatory factors which induce signal transduction within epithelial cells. This interrelationship is important for normal growth regulation. However, it should be noted that human BPH is not associated with elevated DHT levels.[3] The paradox of continuing prostatic growth with declining androgen levels suggests that other factors secreted by the testis can stimulate prostate growth or perhaps sensitize prostatic cells to the effect of androgen.[4] Estrogen may act in combination with other hormones to stimulate prostate cells and cause enlargement of the gland. Estrogen-androgen synergisms as well as a role for other steroidal hormones have also been suggested as mechanisms for BPH.

Estrogens play a role in proliferation in the prostate, but interestingly are capable of stimulating as well as inhibiting growth. This duality of action is specifically due to activation of each ER: ERa and ERβ.

Estrogen action, mediated via ERa, will cause aberrant cellular differentiation and proliferation with progression to prostatic hyperplasia, neoplasia, and dysplasia.[5–7]

Earlier studies[8,9] established that the epithelial and stromal elements both contain AR and 5aR and that the estrogen receptor, ERa, is primarily localized in stromal tissue. Consequently, estrogens have long been implicated in stromal cell hyperplasia and the development of the stromal adenoma that results in the bladder outflow obstruction associated with BPH. Estrogens may exercise a synergistic role with DHT in promoting this effect, but could be more concerned in the suppression of apoptosis. Whatever mechanism obtains, the accumulated studies of Krieg *et al*.[10] support the concept that the age-related metabolism of androgens and estrogens can be considered responsible for the stromal cell hyperplasia associated with BPH. They have shown that estrogen accumulation in the human prostate is an age-dependent event. Analysis of the concentrations of oestradiol-17b and estrone in both compartments of normal and hyperplastic prostates clearly demonstrated an increase in the levels of both estrogens in the stroma with increasing age. The levels in epithelial tissue remained constant. Interestingly, DHT concentrations in the epithelium decreased with increasing age, whereas the levels in stroma remained constant. The overall picture portrays an enhanced estrogenic influence, relative to that of DHT, in the elderly man. Support studies[11] show an age-related decrease in DHT levels in the transition zone (TZ) of the human prostate, contemporaneously with a resultant enhanced estrogen/androgen ratio in this region of the prostate. The influence of both androgens and estrogens on the promotion of smooth muscle hyperplasia seems pivotal to these complex epithelialstromal interactions, relating closely to the classical studies of Walsh and Wilson[12] that highlighted the synergistic role of the hormones in the promotion of prostate growth.

More recently, an antiproliferative action of estrogen was suggested to occur via activation of the epithelial ERβ.[13–15] The aromatase knock-out (ArKO) mouse is estrogen deficient and develops prostatic hyperplasia and hypertrophy,[16] both of which are suppressed and/or ablated in intact animals following the administration of an ERβ-specific (but not ERa) agonist.[17]

Aromatase, the enzyme required for metabolism of androgens to estrogens, is expressed in the stroma of the normal prostate.[18] The absence of aromatase activity in both the stroma and epithelium of prostate tissue recombinants results in epithelial hyperplasia comparable to that seen in intact ArKO mice and in men with benign prostatic hyperplasia.[19] The absence of local aromatase expression in the prostate is a key factor in determining epithelial cell hyperplasia. Aromatase deficiency, and therefore the absence of local estrogen and failure to activate ERβ, locally and within the tissue itself, will result in the development of prostatic hyperplasia and is independent of the systemic hormone status. In the absence of ERβ signaling, increased cell proliferation results in non-neoplastic increases in epithelial tissue identified as epithelial hyperplasia.[19]

Although androgens are essential for the coordinated growth of the prostate, local estrogenic activity is equally essential for the modulation of normal prostate development. Estrogen, acting via ERβ, has a definite antiproliferative effect on prostatic epithelium that is able to prevent and ablate prostatic epithelial hyperplasia. Therefore, estrogens, acting in synergy with androgens and ERβ, are required to regulate the proliferative and antiproliferative changes that occur during normal prostate development and differentiation.

## EPITHELIAL-STROMAL INTERACTIONS

Stromal-epithelial interactions play critical roles in the hormonal, cellular, and molecular regulation of normal and neoplastic prostatic development.[20] Aging permits gradual accumulation of prostate mass as a result of continuing glandular-stromal interactions, which may be enhanced by various growth factors provided systemically via the circulation or locally via the urethra. A study conducted by Cunha *et al*.[21] showed that stromal cells have the ability to modulate the differentiation pattern of normal prostatic epithelium. Other studies have shown that growth factors produced by both the epithelial and stromal cells can regulate the alternate cell type. Aberrant expression of peptide growth factors or their receptors can directly contribute to uncontrolled growth, resulting in BPH. The stromal cells are responsible for secreting many growth factors such as fibroblast growth factors, insulin-like growth factors I and II, as well as tumor growth factors, which act in an autocrine manner on the stroma itself as well as the neighboring glandular cells to induce proliferation.

The androgen-promoted production of KGF (FGF-7) by the fibroblasts of the stroma exercises a mitogenic action on epithelial cells, an effect probably mediated by the FGFR-2exonIIIb receptor. This receptor, a splice variant of FGFR-2, is expressed by epithelial cells and specifically recognizes KGF.[22] TGF-β, produced by stromal smooth muscle cells[23] and considered to be directly concerned in the regulation of apoptosis,[24] inhibits this action of KGF. KGF, however, does not promote stromal-stromal-cell proliferation. FGF-2 promotes an autocrine mitogenic action in the stroma, mediated by the receptor FGF-R1, but has no effect on epithelial cells. FGFR1, encoded by the FGF-R1 (flg) gene, is normally localized only in stroma. The factors which control the growth and differentiation of the stromal cell population are still not well understood. Cultured fibroblasts produce FGF-2. DHT alone has no effect on prostatic fibroblast proliferation, but does in association with FGF-2.

Other components, which are thought to be involved in growth regulation and are secreted by the prostate cells are matrix metalloproteinases (MMPs). The production of MMPs by prostatic epithelial and/or neighboring stromal cells gives cells the capability to penetrate extracellular matrix barriers in normal or neoplastic growth.[25]

BPH involves qualitative and quantitative alterations in extracellular matrix components affecting stromal-epithelial interactions. Glycosaminoglycans (GAGs) are polysaccharide components of the extracellular matrix whose role in the development of BPH is under investigation. The apparent increase in chondroitin sulphate and decrease in dermatan sulphate content in prostates of patients with BPH is in good agreement with the pathological manifestation of increased cell proliferation in hyperplastic prostate tissue, since these glycan molecules have been reported to increase and decrease cell proliferation, respectively.[26]

One of the organs believed to be influenced by the EGF system is the prostate gland. Stimulation with EGF induces proliferation of epithelial cells derived from the prostate. Proliferation of stromal cells from the prostate has also been shown to be increased by growth factor stimulation. In the prostate, the EGF system has also been suggested to play an important role for stroma-epithelium interactions.[27]

The interactions between the stromal and epithelial compartment of the prostate, which are transmitted through the extracellular matrix component, play a very important role in the etiology of BPH. Technological advances in both cellular and molecular biology will help to better elucidate these interactions and their important role in the development of BPH in the near future.

## INFLAMMATORY ASPECTS ASSOCIATED WITH BPH

BPH nodules frequently occur concurrently with chronic inflammatory infiltrates, mainly composed of chronically activated T cells and macrophages.[28–30] Chronic inflammation may have a prominent role in BPH progression.

A causative role for inflammation in the pathogenesis of BPH was first proposed in 1937.[31] However, for the major part of the 20th century, the embryonal reawakening theory[32] dominated the field of BPH. Kohnen and Schaeffer reopened the discussion on the inflammatory nature of BPH 35 years ago.[33,34] Investigations of experimentally induced prostatitis in mice and rats suggested autoimmune responses, age-dependent hormonal imbalances, and genetic background to be causative factors.[35,–38]

Three recent reviews on the pathogenesis of BPH have provided an evidence based thesis that strongly suggests a role of inflammation in the propagation of histological BPH.[37–41] Kramer and Marberger[41] have recently outlined the current state of knowledge in regard to the influence of inflammation on the pathogenesis of BPH. Chronic inflammatory infiltrates, mainly composed of chronically activated T cells and macrophages frequently are associated with BPH nodules.[28–30] These infiltrating cells are responsible for the production of cytokines (IL-2 and IFNγ) which may support fibromuscular growth in BPH.[42] Immigration of T cells into the area is attracted by increased production of proinflammatory cytokines such as IL-6, IL-8, and IL-15.[40,43,44] Surrounding cells become targets and are killed (unknown mechanisms), leaving behind vacant spaces that are replaced by fibromuscular nodules with a specific pattern of a Th0/Th3 type of immune response.[45] What we do not know is why the leukocyte population increases in BPH. A number of hypotheses have been generated based on recent basic research. In-situ studies demonstrated elevated expression of pro-inflammatory cytokines in BPH. IL-6, IL-8, and IL-17 may perpetuate chronic immune response in BPH and induce fibromuscular growth by an autocrine or paracrine loop[45,46] or via induction of COX-2 expression.[47] Immune reaction may be activated via Toll-like receptor signaling and mediated by macrophages and T cells.[46] Conversely, anti-inflammatory factors such as macrophage inhibitory cytokine-1[48] may be decreased in symptomatic BPH tissues. Animal models provided evidence for the presence of unique T-cell subsets which may suppress autoimmunity in healthy Sprague-Dawley rats resistant to chronic nonbacterial prostatitis.[49] Based on the available scientific evidence, that it is highly likely that age-dependent weakening of the immune system, coupled with modified hormonal secretion, leads to the deterioration of a postulated population of suppressor cells that actively suppresses the recognition of prostatic antigens which leads to gradual infiltration of the prostate by lymphocytes and subsequent cascade of events that leads to BPH.[50]

The pathogenesis of BPH is still not certain, although chronic inflammation may have a prominent role in disease progression. Regardless of its reasons, the end effect of chronic inflammation is an atypical cytokinerich milieu, which may lead to alterations in the microenvironment and a chronic repetitive wound healing ending in the development of BPH nodules. Further research is required to determine the putative (auto) antigen, the immune response of patients and a new classification of BPH quantifying local and systemic inflammatory/immune reactions in relation to clinical relevance.[41]

## ROLE OF PROSTATIC VASCULAR SYSTEM IN THE DEVELOPMENT OF BPH

Recent studies in animal models have shown that the prostatic vascular system is also likely to be a primary target of androgen action.[51–53] It has been observed that one of the earliest postcastration physiologic changes in prostatic tissue is a dramatic reduction of blood flow. This change in blood flow was completely dependent upon declining androgen levels in these rats. Severe constriction of the prostatic vessels after castration was likely caused by a rapid decline of prostatic nitric oxide synthase activity, as reported by Hayek *et al*.[54] The mechanistic explanation for the rapid degeneration of the prostate gland capillary bed remains unclear at this time, although it is likely related to a complex change in vascular regulatory factors expressed by the prostate after castration.[55] It suggests that the later occurring loss of the bulk of prostate cells by apoptosis may be driven by the loss of appropriate oxygenation (hypoxia) or nutrition that occurs as a result of the loss of prostatic blood flow. It was proposed that the abnormal prostate growth process associated with human BPH must also be obligatorily accompanied by an angiogenic process providing sufficient nutrition and oxygenation for growing prostate cell survival. If this BPH-associated angiogenic process precedes the increase in prostate mass (as it does in the rat prostate model), then the prostatic vascular system should be considered a target for the development of therapies designed to slow or stop the abnormal prostate growth associated with BPH. Remarkably, this concept may already be apparent in the manner in which certain drug therapies are used to alleviate some of the noxious symptoms associated with BPH. Obviously, any agent that suppresses hematuria acts on the vascular system of the abnormal prostate, thus supporting the concept that the antiandrogenergic effects of finasteride work to suppress blood flow and vascular development in the abnormal prostate gland.

It is very interesting that cardiovascular disease is now being recognized as an important risk factor for BPH.[56,57] Because both diseases manifest in the population as a function of increasing age it would be tempting to think this is merely a correlative association. However, these studies[56,57] segregated age-matched populations into patients with clinical signs of cardiovascular disease/atherosclerosis/hypertension[57] versus those without, and the results indicated that patients with overt vascular pathology were at much higher risk for BPH then those without, regardless of age. Given this new information, it should not be surprising that there have been recent reports that laboratory rats with genetically inbred vascular dysfunctions (spontaneously hypertensive rats or SHR) also appear to develop a condition similar to BPH.[58] These rats were found to develop aging-associated benign proliferative lesions, mainly involving epithelial elements of the prostate gland. These lesions were noted to intensify over time. Likewise, Golomb *et al*.[59] showed that phenylephrine, an α-adrenergic agonist that induces hypertension, also induces atypical glandular BPH in treated rats. It remains to be explained why systemic vascular dysfunctions might specifically affect the prostate gland, a question that may be better answered with more research into the vascular system of the human prostate gland.

The idea was developed that the prostatic vascular system is an important component of prostate growth and regulation, and that dysfunction in blood flow to the prostate gland may be involved in the process through which BPH develops and is controlled.

## ALTERED EXPRESSION OF GENES IN BPH

Gene expression analysis by DNA microarray technology has allowed us to investigate alterations in thousands of genes in diseased versus normal prostatic tissues. Genetic alterations associated with BPH have been an ongoing interest in many laboratories.

Gene expression patterns in BPH are different from normal and cancer. For example in a cluster analysis of more than 6,500 genes by Stamey *et al*. 22 genes were up-regulated

Luo *et al*.[60] reported that a subset of 76 genes involved in a wide range of cellular functions was identified to be differentially expressed between BPH and normal prostate tissues. Genes consistently upregulated in BPH when compared to normal prostate tissues included: a restricted set of growth factors and their binding proteins (e.g., IGF-1 and -2, TGF-beta3, BMP5, latent TGF-beta binding protein 1 and -2); hydrolases, proteases, and protease inhibitors (e.g., neuropathy target esterase, MMP2, alpha-2-macroglobulin); stress response enzymes (e.g., COX2, GSTM5); and extracellular matrix molecules (e.g., laminin alpha 4 and beta 1, chondroitin sulfate proteoglycan 2, lumican). Genes consistently expressing less mRNA in BPH than in normal prostate tissues were less commonly observed and included the transcription factor KLF4, thrombospondin 4, nitric oxide synthase 2A, transglutaminase 3, and gastrin releasing peptide.

In the study conducted by Kulkarni *et al*.,[61] the molecular differentiation in five groups were analyzed by using microarrays. A set of genes comprising mostly ESTs and several genes associated with cell proliferation (for example, calcium/calmodulin-dependent serine kinase, phosphoserine phosphatase, S-phase kinase-associated protein 2 or p45) were significantly up-regulated in the symptomatic BPH group, and a subset of genes including oncogenes and immediate early genes (for example, ras-related protein, v-jun, v-fos, immediate early protein, and early growth response gene) was highly up-regulated in the BPH with cancer group alone. Two subsets of genes including several inflammatory mediators (for example, lymphotoxin beta, immunoglobulins, and chemokine receptors), cytokines, and extracellular matrix-associated molecules (for example, RANTES, osteonectin, lumican) appear to distinguish symptomatic BPH and BPH with cancer as a separate group distinct from the normal or asymptomatic BPH groups. And a cluster of genes including mostly expressed sequence tag (EST) and genes with potential ORFs of unknown function distinguishes the asymptomatic BPH group from all of the others.

The data revealed in these analyses provide potential targets for biomarkers, as well as a method to classify states of the disease. By examining genes that are altered in BPH, a pathway of disease progression or onset may be determined. These analyses provide a provocative insight into the mechanisms, which underlie this highly prevalent disease of men.
